# Short- and Mid-Term Outcomes of Stenting in Patients with Isolated Distal Internal Carotid Artery Stenosis or Post-Surgical Restenosis

**DOI:** 10.3390/jcm11195640

**Published:** 2022-09-24

**Authors:** Dat Tin Nguyen, Ákos Bérczi, Balázs Bence Nyárády, Ádám Szőnyi, Márton Philippovich, Edit Dósa

**Affiliations:** 1 Heart and Vascular Center, Semmelweis University, 1122 Budapest, Hungary; 2 Hungarian Vascular Radiology Research Group, 1122 Budapest, Hungary

**Keywords:** internal carotid artery, atherosclerosis, restenosis, stenting, outcome, stroke, in-stent restenosis, patency, mortality, survival

## Abstract

The aim was to evaluate the outcome of stenting in patients with isolated distal internal carotid artery (ICA) stenosis or post-surgical restenosis, as no data are currently available in the literature. Sixty-six patients (men, N = 53; median age: 66 [IQR, 61–73] years) with ≥50% distal ICA (re)stenosis were included in this single-center retrospective study. The narrowest part of the (re)stenosis was at least 20 mm from the bifurcation in all patients. Patients were divided into two etiological groups, atherosclerotic (AS, N = 40) and post-surgical restenotic (RES, N = 26). Postprocedural neurological events were observed in two patients (5%) in the AS group and in two patients (7.7%) in the RES group. The median follow-up time was 40 (IQR, 18–86) months. Three patients (7.5%) in the AS group had an in-stent restenosis (ISR) ≥ 50%, but none in the RES group. Three patients (7.5%) in the AS group and seven patients (26.9%) in the RES group died. None of the deaths in the RES group were directly related to stenting itself. The early neurological complication rate of stenting due to distal ICA (re)stenoses is acceptable. However, the mid-term mortality rate of stenting for distal ICA post-surgical restenoses is high, indicating the vulnerability of this subgroup.

## 1. Introduction

The most common sites of atherosclerotic lesions of the carotid arteries are the bifurcation, the 10–15 mm proximal segment of the internal carotid artery (ICA), and the origin/proximal third of the left common carotid artery (CCA). Atherosclerosis rarely affects the distal part of the ICA [1]. Invasive therapy for atherosclerotic carotid stenoses includes open surgery, stenting, or a combination of both [2,3]. Distal ICA lesions can only be approached with great difficulty by open surgery, either from the retromandibular fossa or by other means (e.g., the mobilization of the parotid gland, double mandibular osteotomy, or mandibular subluxation with styloidectomy) [4,5,6,7]. For this reason, stenting rather than open surgery is the invasive option for these patients, even for those who are symptomatic [2].

After open carotid surgery, restenosis occurs in 0.3–9% of cases [8]. Like atherosclerotic stenoses, restenoses can localize to the distal ICA [9]. Stenting is also the main invasive therapy for distal ICA restenoses [2].

Since there are no literature data on the short- and mid-term efficacy of stenting in atherosclerotic or post-surgical restenotic distal ICA stenoses, our aim was to provide information on this topic.

## 2. Materials and Methods

### 2.1. Study Design

This single-center retrospective study analyzed patients (N = 66) who underwent stenting for atherosclerotic or post-surgical restenotic isolated distal ICA stenosis between January 2001 and January 2020.

### 2.2. Stenting Process

For each patient, the vascular team at our center decided on the need for stenting based on the European Society for Vascular Surgery guidelines in force at the time. Patients were considered symptomatic if they had any ischemic neurological event (amaurosis fugax, transient ischemic attack [TIA], minor or major stroke) in the ipsilateral carotid territory within 6 months before the intervention [10].

Stenting was performed in the standard manner [11] by three interventional radiologists with more than 10 years of experience, with the implantation of self-expanding stents and embolic protection. If the patient was not on antiplatelet therapy or was on monotherapy only, 100–300 mg acetylsalicylic acid and/or 75 mg clopidogrel daily was started at least 72 h before the procedure. In urgent cases, a loading dose of 250–500 mg acetylsalicylic acid and/or 300–600 mg clopidogrel was given. In the absence of cardiological or other indications, dual antiplatelet therapy was continued for 1 month after the intervention, followed by monotherapy for an indefinite period [12].

Stenting was technically successful if no extravasation, dissection, or >30% residual stenosis was seen on final digital subtraction angiography (DSA) images [11].

### 2.3. Follow-Up Visits

Follow-up visits were scheduled for the 6th week after the intervention, the 6th and 12th month, and then once a year. However, due to complaints, contralateral invasive carotid procedure, or other reasons, these dates could be changed. Follow-up visits consisted of interviewing the patient and ultrasound examination of the cervical arteries. Restenosis was defined as 50–69% if the peak systolic velocity (PSV) inside the stent or at either end of the stent was 225–350 cm/s and ≥ 70% if PSV was >350 cm/s [13]. If the distal part of the stent was not visible by ultrasound but indirect signs (ICA flow volume <159 mL/min, ICA PSV < 33 cm/s, and/or CCA PSV < 42 cm/s) suggested a ≥70% in-stent restenosis (ISR) [14], the patient was submitted to computed tomography angiography (CTA).

### 2.4. Analyzed Parameters

Cardiovascular risk factors and comorbidities (female sex, age ≥ 80 years, hypertension, hyperlipidemia, diabetes mellitus, and chronic kidney disease), previous invasive vascular therapies, lesion- and intervention-related parameters, neurological events (amaurosis fugax, TIA, minor or major stroke) before and after the stenting, ISR characteristics and primary patency and mortality rates were assessed. For a definition of cardiovascular risk factors and comorbidities, see another publication by our research group [15]. The parameters of the lesions (localization, grade and length of stenosis, presence, and severity of calcification) were determined on preprocedural CTA scans. By localization, the affected side and the distance of the narrowest part of the ICA stenosis from the bifurcation was meant. The percentage of stenosis was calculated using the formula in the North American Symptomatic Carotid Endarterectomy Trial [16]. The length of the lesion was defined as the distance between the proximal and distal points where the grade of stenosis decreased to 80% of its maximum [17]. The severity of calcification was classified according to Woodcock and four types, such as absent, mild (thin, discontinuous), moderate (thin, continuous or thick, discontinuous), and severe (thick, continuous), were distinguished [18]. Among the intervention-related parameters, the puncture site, the type of embolic protection device, the manufacturer, diameter and length of the balloons and stents, and the complications were collected. Regarding the definition of neurological events, reference is made to a guideline [19]. ISR characteristics included the ultrasonographic grade, localization (in-stent, peristent, or both), and pattern (focal or diffuse) of restenotic lesions. The ISR was considered focal if it was shorter than 10 mm. Primary patency was defined as freedom from ≥50% ISR or occlusion.

### 2.5. Statistical Analysis

Statistical analysis was performed using SPSS Statistics for Windows (Version 25.0.; IBM Corp., Armonk, NY, USA) and GraphPad Prism 7.01 (GraphPad Software Inc., La Jolla, CA, USA) software. Continuous data were presented as median and interquartile range (IQR) and compared using Mann–Whitney *U* test. Categorical data were expressed as numbers (percentages) and compared using Fisher’s exact test. Kaplan–Meier analysis was performed to determine primary patency and mortality rates. Follow-up was maximized at 48 months. Survival curves were compared using a log-rank test. All statistical tests were two-tailed. The threshold for statistical significance was *p* ≤ 0.05.

## 3. Results

### 3.1. Patient Data

The median age of the 66 patients (women, N = 13; men, N = 53) was 66 years (IQR, 61–73 years). Patients were divided into two etiological groups, atherosclerotic (AS, N = 40 [60.6%]; median age: 67 years [IQR, 61–74 years]) and post-surgical restenotic (RES, N = 26 [39.4%]; median age: 64.5 years [IQR, 60.5–71 years]). There was no significant difference (*p* = 0.541) in median age between the AS and RES groups. The carotid surgery in all patients was an eversion endarterectomy. The median time between carotid surgery and stenting was 80 months (IQR, 22–148 months). Stenting was carried out in nine patients within 48 months after endarterectomy. Patient-related parameters are shown in Table 1. Of the 66 patients, 15 (22.7%) had some neurological symptoms before stenting. There was no significant difference (*p* > 0.999) in preprocedural neurological events between the two groups. The RES group had significantly more women (*p* = 0.003) and significantly more patients with hypertension (*p* = 0.010), contralateral carotid invasive treatment (*p* = 0.015), and lower extremity arterial reconstruction (*p* = 0.046).

### 3.2. Lesion Data

Lesion characteristics can be found in Table 2. The narrowest part of the ICA stenosis was at least 20 mm from the bifurcation in all patients. Among lesion-related parameters, only length was significantly different between the two groups; AS lesions were significantly longer (*p* = 0.002) than RES lesions.

### 3.3. Procedure Data

In the AS group, the access site was femoral in 28 cases (70%) and radial in 12 cases (30%), while in the RES group, the access site was femoral in 13 cases (50%), radial in 10 cases (38.5%), and brachial in three cases (11.5%). In the AS group, embolic protection was distal type (FilterWire EZ; Boston Scientific Corp., Marlborough, MA, USA) in 38 patients (95%) and proximal type (Mo.Ma; Medtronic Inc., Minneapolis, MN, USA) in two patients (5%). In the RES group, all patients had distal type embolic protection. Six cases (15%) in the AS group and one case (3.8%) in the RES group required predilation. Five different types of self-expanding stents were used. Twenty-eight (42.4%) of the stents were located only in the ICA and did not extend into the bifurcation and CCA. All stents were postdilated. Technical success was achieved in 100% of cases. The types, diameters, and lengths of balloons and stents are listed in Table 3.

### 3.4. Early (≤30 Days) Postprocedural Period

There were five intervention-related complications: one inguinal haematoma (1.5%) not requiring evacuation and four neurological events (6.1%; AS group, one TIA and one major stroke; RES group, two TIAs). The parameters of patients with postprocedural neurological complications can be seen in Table 4. TIAs presented as contralateral upper and/or lower limb paresis or dysarthria and lasted no longer than 15 min. None of the TIA patients had an acute ischemic or hemorrhagic brain lesion on post-stenting CT or magnetic resonance images. The time between carotid surgery and stenting was 103 months in Patient 3 and 178 months in Patient 4. The major stroke patient became unconscious 2 h after an uneventful stenting procedure. The emergency CT scan showed extensive bleeding in the ipsilateral frontal and parietal lobes. The patient died on day 37 after stenting.

### 3.5. Follow-Up Period

**Figure 1:**
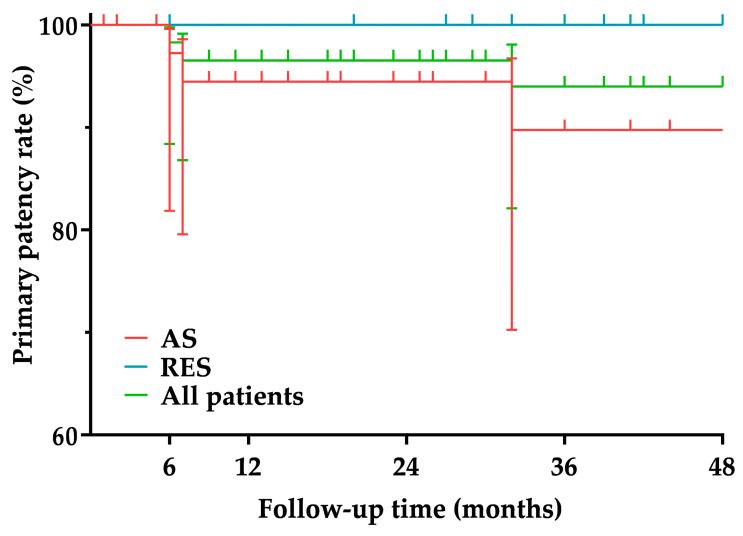
Primary patency. AS, Atherosclerotic; RES, restenotic.

**Figure 2:**
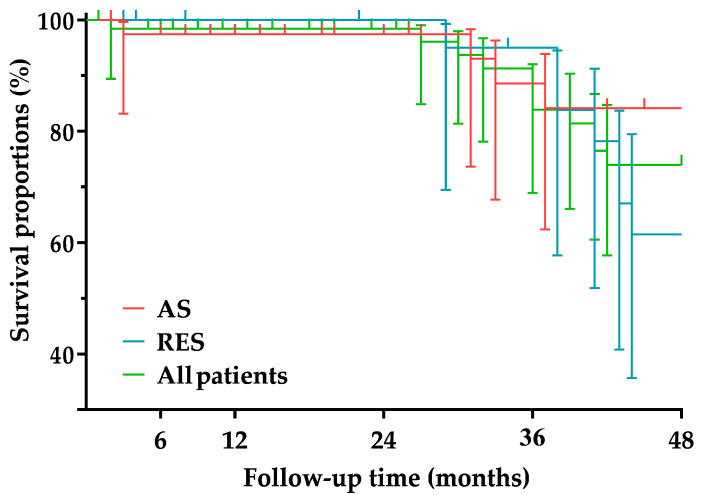
Survival proportions. AS, Atherosclerotic; RES, restenotic.

The median follow-up time was 34 months (IQR, 15–87 months) in the AS group and 41 months (IQR, 28–74 months) in the RES group. There was no significant difference (*p* = 0.708) in follow-up time between the two groups. In the AS group, two cases (5%) of 50–69% ISR and one case (2.5%) of ≥70% ISR were detected. All ISRs were located within the stent and were of the focal type. Patients with ISR were asymptomatic. The patient with ≥70% ISR underwent reintervention with a plain balloon (Trek; Abbott Vascular Inc., Santa Clara, CA, USA; size, 4 mm × 20 mm). No one in the RES group had ISR. The primary patency rate was 97.2% at 6 months, 94.4% at 12 and 24 months, and 89.7% at 36 and 48 months in the AS group and 100% over the entire follow-up period in the RES group. The primary patency rates of the two groups were not significantly different (*p* = 0.528) (Figure 1 and Table 5). During follow-up, three patients (7.5%) in the AS group and seven patients (26.9%) in the RES group died. The cause of death was myocardial infarction in three patients, heart failure in two patients, malignancy in two patients, major stroke in one patient, chronic obstructive pulmonary disease in one patient, and gastrointestinal bleeding in one patient. The survival proportion was 97.4% at 6, 12, and 24 months and 84.1% at 36 and 48 months in the AS group and 100% at 6, 12, and 24 months, 83.8% at 36 months, and 61.5% at 48 months in the RES group. The survival proportions of the two groups were not significantly different (*p* = 0.289) (Figure 2 and Table 6).

## 4. Discussion

Most studies have separately analyzed the short- and mid-/long-term outcomes of carotid artery stenting (CAS) for atherosclerosis and post-surgical restenosis [19,20], but we found eight studies that did so comparatively [21,22,23,24,25,26,27,28]. The two main indicators of the short-term success of CAS are the rate of new or recurrent neurological events and mortality. The rate of stroke within 30 days after stenting ranges from 0% to 9.8%, while the rate of all-cause mortality within 30 days after stenting ranges from 0% to 1.3% for atherosclerotic ICA stenoses [19]. The same rates for post-surgical ICA restenosis stenting range from 0% to 18% and 0% to 2%, respectively [20]. In three of the eight comparative studies, peri- and postprocedural neurological complications were more frequent in patients undergoing stenting for atherosclerotic ICA stenoses [22,23,24]. The other five studies showed no significant difference in neurological events within 30 days after stenting between the two etiological groups [21,25,26,27,28]. The etiology of ICA lesions had no effect on CAS 30-day mortality in any of the comparative studies [21,22,23,24,25,26,27,28]. However, to the best of our knowledge, no study has specifically investigated the outcome of CAS in distal ICA lesions. In our AS group, the rate of neurological complications within 30 days after stenting was 5%, resulting from one TIA and one hemorrhagic stroke; the patient with hemorrhagic stroke died on day 37 after the intervention. The underlying cause of the hemorrhagic stroke was presumably hyperperfusion syndrome. After CAS, hyperperfusion syndrome occurs in 0–21.2% of cases and consequential hemorrhagic stroke in 0–100% of cases [29]. In our RES group, compared to our AS group, a non-significantly higher proportion of patients, 7.7%, developed neurological symptoms within 30 days after stenting, but no deaths were recorded in the early postprocedural period. Thus, the short-term success rates for stenting distal ICA (re)stenoses are not worse than the rates reported for stenting ICA (re)stenoses in general (without defining the lesion location).

The mid-/long-term outcome of CAS is best characterized by ISR and mortality rates. In some publications, both the ISR ≥ 50% rate and the ISR ≥ 70% rate are given [30,31,32], while in others, only the ISR ≥ 70% rate is mentioned [33,34,35,36,37,38]. Based on literature data, the prevalence of ISR ≥ 50% after stenting for atherosclerotic ICA lesions is between 0% and 37% [30,31,32], while the prevalence of ISR ≥ 70% is between 0% and 9.8% [33,34,35,36,37,38]. For post-surgical ICA restenosis stenting, these incidences range from 0% to 15% [39,40] and 0% to 9.5% [41,42], respectively. Of the eight comparative studies, only two examined ISR (one considered ISR ≥ 50% [21], the other considered ISR ≥ 70% as the endpoint [26]), and none revealed a significant difference in the prevalence of ISR between the two etiological groups [21,26]. In our patient population, the incidence of ISR was non-significantly higher in the AS group (ISR ≥ 50%, 7.5% and ISR ≥ 70%, 2.5%) than in the RES group (ISR ≥ 50%, 0% and ISR ≥ 70%, 0%). Thus, the ISR rates for stenting distal ICA (re)stenoses (such as the short-term results) are not worse than the rates reported for stenting ICA (re)stenoses in general (without defining the lesion location).

Only a few publications were found that included mid-/long-term mortality rates for CAS. For CAS performed for atherosclerotic ICA stenoses, the mid-/long-term mortality rate ranges from 12.1% to 35% [18,20,30,32,42,43], while for CAS performed for post-surgical ICA restenoses, the mid-/long-term mortality rate ranges from 9.6% to 11.8% [20,41,44]. Of the eight comparative studies, only one study aimed to determine the mid-term (4-year) mortality rate [21]. In this study, there was no significant difference in the 4-year mortality rate between CAS for atherosclerosis (12.1%) and CAS for post-surgical restenosis (11.8%) [21]. The mid-term mortality rate of 7.5% in our AS group is low, while the mid-term mortality rate of 26.9% in our RES group is quite high in light of the literature. It is important to note, however, that none of the deaths in our RES group were directly related to CAS itself; the deaths were the result of other serious comorbidities in the patients.

Our study has two main limitations: its retrospective nature and the relatively small number of patients.

## 5. Conclusions

The early complication and ISR rates of distal ICA stenting are acceptable and are not influenced by the etiology of the lesion. However, the mid-term mortality rate of the RES group is high. The lower survival is probably not due to the stenting procedure but rather to the more complex comorbidity profile of the RES population.

## Figures and Tables

**Table 1:** Preprocedural neurological events, cardiovascular risk factors, comorbidities, and previous invasive vascular therapies.

Patient-Related Parameters	AS Group (N = 40)	RES Group (N = 26)	*p*-Value
Preprocedural neurological events, N (%)	9 (22.5)	6 (23.1)	>0.999
Amaurosis fugax, N (%)	3 (7.5)	0 (0)	0.273
TIA, N (%)	3 (7.5)	6 (23.1)	0.139
Minor stroke, N (%)	3 (7.5)	0 (0)	0.273
CV risk factors, comorbidities			
Female sex, N (%)	3 (7.5)	10 (38.5)	0.003
Age ≥ 80 years, N (%)	5 (12.5)	2 (7.7)	0.695
Hypertension, N (%)	28 (70)	25 (96.2)	0.010
Hyperlipidemia, N (%)	13 (32.5)	8 (30.8)	>0.999
Diabetes mellitus, N (%)	8 (20)	8 (30.8)	0.384
Chronic kidney disease, N (%)	2 (5)	4 (15.4)	0.202
Previous invasive vascular therapies			
Coronary artery invasive treatment, N (%)	11 (27.5)	3 (11.5)	0.217
Contralateral carotid artery invasive treatment, N (%)	8 (20)	13 (50)	0.015
Subclavian artery invasive treatment, N (%)	0 (0)	1 (3.8)	0.394
Visceral artery invasive treatment, N (%)	1 (2.5)	2 (7.7)	0.557
Aortic invasive treatment, N (%)	0 (0)	1 (3.8)	0.394
Lower extremity arterial invasive treatment, N (%)	7 (17.5)	11 (42.3)	0.046

AS, Atherosclerotic; CV, cardiovascular; N, number; RES, restenotic; TIA, transient ischemic attack.

**Table 2:** Lesion characteristics.

Lesion-Related Parameters	AS Group (N = 40)	RES Group (N = 26)	*p*-Value
Right side, N (%)	15 (37.5)	14 (53.8)	0.214
Distance from the bifurcation (mm), median (IQR)	20.4 (20.1–21.4)	21.5 (20.1–24)	0.126
Stenosis grade (%), median (IQR)	90 (80–90)	90 (85–95)	0.099
Stenosis length (mm), median (IQR)	8.1 (6.1–12)	5.1 (4.1–7.5)	0.002
Calcification, N (%)	25 (62.5)	11 (42.3)	0.133
Mild, N (%)	14 (35)	8 (30.8)	0.794
Moderate, N (%)	8 (20)	1 (3.8)	0.077
Heavy, N (%)	3 (7.5)	2 (7.7)	>0.999

AS, Atherosclerotic; IQR, interquartile range; N, number; RES, restenotic.

**Table 3:** Balloon and stent characteristics.

Balloon- and Stent-Related Parameters	AS Group (N = 40)	RES Group (N = 26)
Predilation balloons		
Maverick (Boston Scientific Corp., Marlborough, MA, USA), N (%)	4 (10)	0 (0)
Emerge (Boston Scientific Corp.), N (%)	1 (2.5)	0 (0)
Pantera Pro (Biotronik SE & Co. KG, Berlin, Germany), N (%)	1 (2.5)	0 (0)
Sprinter Legend Rx (Medtronic Inc., Minneapolis, MN, USA), N (%)	0 (0)	1 (3.8)
Diameter (mm), range	2.5–4	2.5
Length (mm), range	20–40	12
Postdilation balloons		
Sterling (Boston Scientific Corp.), N (%)	25 (62.5)	12 (46.2)
Maverick (Boston Scientific Corp.), N (%)	8 (20)	5 (19.2)
Viatrac 14 Plus (Abbott Vascular Inc., Santa Clara, CA, USA), N (%)	5 (12.5)	6 (23.1)
Ultra-Soft SV (Boston Scientific Corp.), N (%)	2 (5)	3 (11.5)
Diameter (mm), range	4–6	4–5
Length (mm), range	20–40	20–40
Stents		
Wallstent (Boston Scientific Corp.), N (%)	32 (80)	25 (96.2)
Xact (Abbott Vascular Inc.), N (%)	4 (10)	0 (0)
Roadsaver (Terumo Corp., Tokyo, Japan), N (%)	1 (2.5)	1 (3.8)
Precise Pro Rx (Cordis Corp., Johnson & Johnson Co., Miami, FL, USA), N (%)	2 (5)	0 (0)
Exponent (Medtronic Inc.), N (%)	1 (2.5)	0 (0)
Diameter (mm), range	5–9	5–9
Length (mm), range	25–50	30–50

AS, Atherosclerotic; N, number; RES, restenotic.

**Table 4:** Parameters of patients with postprocedural neurological complications.

Parameters	Patient 1 with Postprocedural TIA	Patient 2 with Postprocedural Major Stroke	Patient 3 with Postprocedural TIA	Patient 4 with Postprocedural TIA
Sex	Male	Male	Female	Female
Age	59 years	87 years	67 years	86 years
Etiological group	AS	AS	RES	RES
Preprocedural symptom	No	TIA	Minor stroke	TIA
Contralateral ICA stenosis/occlusion	Occlusion	No	Stenosis	Stenosis
Ipsilateral preprocedural stenosis grade	90%	95%	90%	95%
Ipsilateral preprocedural stenosis length	6.2 mm	16.8 mm	3.3 mm	4.5 mm
Calcification	Mild	Absent	Absent	Mild
Predilation	No	Yes	No	No
Stent type	Wallstent	Wallstent	Wallstent	Wallstent
Postprocedural ultrasound	Patent stent	Patent stent	Patent stent	Patent stent

AS, Atherosclerotic; RES, restenotic; TIA, transient ischemic attack.

**Table 5:** Primary patency.

		6 Months	12 Months	24 Months	36 Months	48 Months
All patients	%	98.3	96.5	96.5	94	94
	95% CI	88.4–99.7	86.8–99.1	86.8–99.1	82.1–98.1	82.1–98.1
	Number at risk	58	52	45	35	26
AS group	%	97.2	94.4	94.4	89.7	89.7
	95% CI	81.9–99.6	79.5–98.5	79.5–98.5	70.2–96.7	70.2–96.7
	Number at risk	36	31	25	18	16
RES group	%	100	100	100	100	100
	95% CI	-	-	-	-	-
	Number at risk	22	22	21	17	11

AS, Atherosclerotic; CI, confidence interval; RES, restenotic.

**Table 6:** Survival proportions.

		6 Months	12 Months	24 Months	36 Months	48 Months
All patients	%	98.4	98.4	98.4	83.9	73.9
	95% CI	89.4–99.7	89.4–99.7	89.4–99.7	68.9–92.1	55.7–84.7
	Number at risk	58	54	46	37	28
AS group	%	97.4	97.4	97.4	84.1	84.1
	95% CI	83.1–99.6	83.1–99.6	83.1–99.6	62.3–93.8	62.3–93.8
	Number at risk	37	33	26	20	18
RES group	%	100	100	100	83.8	61.5
	95% CI	-	-	-	57.7–94.5	35.7–79.5
	Number at risk	22	22	21	17	11

AS, Atherosclerotic; CI, confidence interval; RES, restenotic.

## Data Availability

The data presented in this study are available on request from the corresponding author. The data are not publicly available due to reasons pertaining to patient privacy.
